# Role of Notch signaling in regulating innate immunity and inflammation in health and disease

**DOI:** 10.1007/s13238-016-0250-0

**Published:** 2016-03-02

**Authors:** Yingli Shang, Sinead Smith, Xiaoyu Hu

**Affiliations:** School of Medicine and Institute for Immunology, Tsinghua University, Beijing, 100084 China; Department of Clinical Medicine, Trinity College Dublin, Dublin, 2 Ireland

**Keywords:** inflammation, innate immunity, macrophages, Notch signaling, RBP-J

## Abstract

The Notch signaling pathway is conserved from *Drosophila* to mammals and is critically involved in developmental processes. In the immune system, it has been established that Notch signaling regulates multiple steps of T and B cell development in both central and peripheral lymphoid organs. Relative to the well documented role of Notch signaling in lymphocyte development, less is known about its role in regulating myeloid lineage development and function, especially in the context of acute and chronic inflammation. In this review article, we will describe the evidence accumulated during the recent years to support a key regulatory role of the Notch pathway in innate immune and inflammatory responses and discuss the potential implications of such regulation for pathogenesis and therapy of inflammatory disorders.

## INTRODUCTION OF THE NOTCH SIGNALING PATHWAY

The evolutionary conserved Notch signaling pathway regulates cell proliferation, apoptosis and cell fate decisions during development and adult tissue homeostasis (Radtke et al., 2010). In mammalian cells there are four Notch receptors (Notch 1–4), that are large single-pass type I transmembrane proteins involved in transducing specific extracellular signals to the nucleus in response to ligand binding. Following translation from a single mRNA transcript, the Notch protein is proteolytically cleaved at site S1 by a furin-like convertase in the Golgi complex and subsequently reassembled into the functional heterodimeric receptor present at the cell surface. The resulting Notch molecule consists of the extracellular domain non-covalently associated with the transmembrane and intracellular domains. The extracelluar domain of all Notch proteins contains 29–36 tandem epidermal growth factor (EGF)-like repeats that mediate ligand interaction, followed by 3 cysteine-rich LIN12 repeats that prevent ligand-independent activation. A hydrophobic stretch of amino acids mediates the heterodimerization between the extracellular, and the transmembrane plus intracellular subunits of the Notch receptor. The intracellular region harbors multiple conserved functional elements including nuclear localization signals, a RAM (RBP-J-association module) domain, ankyrin repeats involved in protein interactions, a trans-activation domain and a C-terminal PEST (praline/glutamic acid/serine/threonine) domain that regulates protein stability (Kopan and Ilagan, 2009).

Canonical Notch signaling is initiated by the binding of the extracellular domain of the Notch receptor to Notch ligands on neighboring cells. In mammals there are five Notch ligands (Jagged1, Jagged2, Delta-like 1 (DLL1), DLL3, and DLL4). Cell type-specific and spatial expression of ligands and/or receptors can regulate Notch signaling to a certain cell context or population (Radtke et al., 2010). Additionally, different ligands and receptors are subject to regulation by other cell signaling pathways triggered by a variety of stimuli (Kopan and Ilagan, 2009). Ligation of Notch receptors by their ligands leads to a sequence of proteolytic events. Firstly, a disintegrin and metalloprotease (ADAM) proteases cleave the receptor at site S2 to release the extracellular domain, which is subsequently endocytosed by the ligand-expressing cell. Following shedding of the extracellular domain, a second cleavage event occurs at site S3 within the transmembrane domain mediated by the multicomponent protease γ-secretase, resulting in the release of the Notch intracellular domain (NICD). NICD is subsequently translocated to the nucleus where it interacts via its RAM domain with the DNA-binding protein recombinant recognition sequence binding protein at the Jκ site (RBP-J, also named CSL or CBF1). In the absence of NICD, RBP-J is bound to specific DNA binding sites ((C/T)GTGGAA) and is thought to act as a transcriptional repressor due to its ability to bind transcriptional corepressors (NCoRs) and histone deacetylases (HDACs). Binding of NICD displaces co-repressor complexes and recruits co-activators including mastermind proteins (MAML1–3), which in turn recruit transcription activation complex in order to induce transcription of Notch target genes (Kopan and Ilagan 2009). Thus RBP-J serves as the nuclear mediator of canonical Notch signaling. Following transcriptional regulation of target genes, NICD is degraded in the nucleus by the ubiquitin-proteasome system. In mammals, the best-described canonical Notch target genes are members of the basic-helix-loop-helix transcription factors belonging to the hairy and enhancer of split (Hes) and hairy and enhancer of split with YRPW motif (Hey) families, including Hes1, Hes5, and Hes7 as well as Hey1, Hey2, and HeyL (Iso et al., 2003). Additional direct Notch target genes include *Deltex1* (Izon et al., 2002), *Il2ra*, *Gata3*, and *Myc* (Borggrefe and Oswald, 2009). Recent studies using global expression analysis and chromatin immunoprecipitation deep-sequencing (ChIP-seq) have revealed genome-wide Notch-RBP-J targets in various systems including hematopoiesis (Hamidi et al., 2011), Epstein-Barr virus infection (Zhao et al., 2011), T-lymhoblastic leukemia/lymphoma (Palomero et al., 2006; Wang et al., 2011), and macrophages (Xu et al., 2015).

One of the most established functions for Notch signaling in the immune system is the differentiation of lymphoid T and B cell lineages (Tanigaki and Honjo, 2007), as well as T cell activation (Eagar et al., 2004), regulatory T cell function (Ostroukhova et al., 2006) and T helper cell differentiation (Amsen et al., 2007; Amsen et al., 2004; Fang et al., 2007; Maillard et al., 2005; Osborne and Minter, 2007; Skokos and Nussenzweig, 2007). These lymphoid-related functions associated with Notch signaling have recently been reviewed (Radtke et al., 2010; Yuan et al., 2010; Yashiro-Ohtani et al., 2010). Less well characterized, however, is the role of Notch signaling in innate immune cell development and function. This review aims to discuss recent findings elucidating a key role for Notch signaling in differentiation, activation and function of the myeloid cells involved in innate immunity and inflammation. First, we will present evidence supporting the notion that active Notch signaling is associated with a variety of inflammatory conditions. Next, we will summarize the current knowledge on regulation of myeloid cell differentiation and function by the Notch pathway. Finally, we discuss the involvement of the Notch pathway in human inflammatory and autoimmune diseases and the potential of targeting Notch signaling as a new approach to modulating inflammation.

## ACTIVE NOTCH SIGNALING UNDER INFLAMMATORY CONDITIONS

Recently, evidence has been mounting that Notch signaling is associated with innate immunity and inflammation. To date, active Notch signaling has been observed under a variety of inflammatory conditions including rheumatoid arthritis (RA) (Nakazawa et al., 2001a; Ando et al., 2003; Jiao et al., 2010; Yabe et al., 2005; Ishii et al., 2001; Nakazawa et al., 2001b; Park et al., 2015), systemic lupus erythematosus (SLE) (Murea et al., 2010; Zhang et al., 2010), atherosclerosis (Fung et al., 2007; Aoyama et al., 2009), systemic sclerosis (Dees et al., 2011), primary biliary cirrhosis (Shackel et al., 2001), preterm labor (Jaiswal et al., 2015), as well as during bacterial and viral infections (Narayana and Balaji 2008; Ito et al., 2009; Ito et al., 2011). Given the recent identification of *RBPJ*, a gene encoding a key nuclear mediator of the canonical Notch pathway, as one of the new RA risk loci (Stahl et al., 2010), association of active Notch signaling with RA is of particular interest. Expression of Notch receptors and ligands were detected in the RA synovial tissues (Nakazawa et al., 2001; Ando et al., 2003; Yabe et al., 2005; Ishii et al., 2001) and aberrant activation of Notch1 was observed in primary synoviocyte cultures from RA patients (Nakazawa et al., 2001). Thus, there is compelling evidence suggesting that the Notch pathway is activated in RA and may modulate disease activities.

Although association of active Notch signaling with inflammatory conditions is supported by a growing body of literature, the mechanisms by which infection and inflammation modulate Notch signaling remain poorly understood. Under an inflammatory environment, it is conceivable that Notch signaling in myeloid cells could be promoted by stimuli that are broadly categorized into two groups: exogenous agents such as pathogens and/or endogenous factors such as cytokines. In the following sections, we will discuss the current knowledge on regulation of Notch signaling by TLR ligands and by inflammatory cytokines respectively.

### Regulation of Notch signaling by TLRs

**Figure 1:**
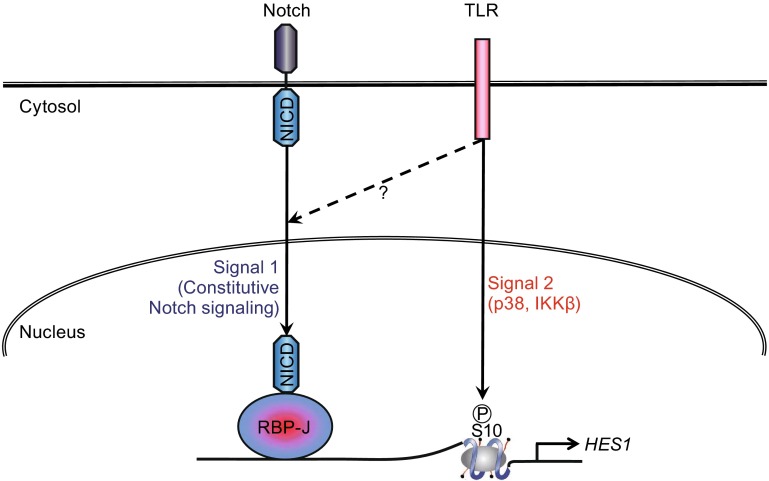
A model for activation of Notch target gene expression in human macrophages. Both signal 1 and signal 2 are required to achieve full-fledged induction of Notch target genes by stimuli such as TLR ligands. Signal 1 is provided by constitutive tonic Notch signaling in macrophages presumably as a result of macrophage-macrophage or macrophage-stromal cell interaction. Signal 2 is provided by TLR stimulation in the form of p38-mediated phosphorylation of histones at the Notch target gene loci

Macrophages and dendritic cells (DCs) express a variety of pattern recognition receptors (PRRs) including toll-like receptors (TLRs) that enable them to rapidly respond to pathogen infections and to coordinate innate and adaptive immune responses. Meanwhile, macrophages and DCs also constitutively express Notch ligands and receptors on their cell surface and thus have the capacity to both induce and respond to Notch signals. One mechanism by which TLRs modulate Notch signaling is by inducing Notch receptor and ligand expression. There is amble evidence that activation of macrophages and DCs with TLR ligands leads to induction of Notch receptors and ligands including Jagged1, DLL1, and DLL4 (Amsen et al., 2004; Fung et al., 2007; Foldi et al., 2010; Monsalve et al., 2006; Monsalve et al., 2009; Palaga et al., 2008; Zhang et al., 2012). Induction of Notch receptor and ligands by purified or synthetic TLR ligands is further confirmed by the experiments using bacterial and viral pathogens such as *Mycobacterium bovis* Bacille Calmette-Guerin (BCG) and influenza H1N1 virus (Narayana and Balaji, 2008; Ito et al., 2009; Ito et al., 2011). Via augmenting expression of Notch receptors and/or ligands, TLR signaling indirectly promotes Notch pathway activation and expression of canonical Notch target genes in a manner that is predicted to be dependent on *de novo* protein synthesis. In addition to the above described indirect activation, we have shown that in human primary macrophages, activation of Notch target genes such as Hes1 and Hey1 can be directly induced by TLR stimulation (Hu et al., 2008). The current observations regarding direct activation of Notch target genes by TLRs support a binary model where signal 1 is provided by tonic Notch signaling and signal 2 is provided by acute TLR signaling (Fig. 1). This binary model is consistent with the following results: (1) As a result of constitutive expression of Notch receptors and ligands, resting macrophages display tonic Notch signaling evidenced by basal levels of NICD. (2) Once triggered by TLR stimulation, activation of Notch target gene expression occurs rapidly in the absence of new protein synthesis, circumventing the requirement for activation secondary to receptor or ligand induction. (3) Signal 1 or signal 2 alone is necessary but not sufficient for full fledged Notch target gene expression in macrophages. Cooperation of both signaling pathways is required for optimal activation. In summary, recent work from a number of laboratories suggests that activation of Notch target genes is a common feature of TLR responses and can occur via two non-mutually exclusive mechanisms, direct activation by acute TLR signaling and indirect activation secondary to Notch receptor and ligand induction. However, a key question that remains unanswered is the identity of the signal that couples acute TLR signaling to Notch pathway activation. Detailed biochemical analysis of Notch pathway components upon TLR stimulation may help solve this issue and yield further insight into the mechanisms of Notch-TLR crosstalk. Another question is the source of so-called “tonic” Notch signaling in myeloid cells. The Notch pathway can be activated in macrophages and DCs *in vivo* by Notch ligands that are expressed by macrophages and DCs themselves, and also by Notch ligands expressed on stromal and epithelial cells in the marginal zone of the spleen, thymic epithelium and bone marrow stromal cells, or on stromal cells at inflammatory sites such as rheumatoid arthritis synovium (Tanigaki and Honjo, 2007; Caton et al., 2007). Future studies utilizing the Notch activity reporter system *in vivo* might be useful in pinpointing the source of Notch ligands under inflammatory conditions.

### Regulation of Notch signaling by inflammatory cytokines

In the section above, we have discussed regulation of the Notch pathway by agents that are foreign to our bodies such as TLR ligands. In this section, we will extend the discussion to regulation of Notch signaling by endogenous factors that are highly involved in immune regulation. Inflammatory cytokines such as TNF and interlukin-1β (IL-1β) are abundantly present during the course of innate immune and inflammatory responses and are essential for host defense against a variety of pathogens. However, under conditions of uncontrolled inflammation and in autoimmune diseases, dysregulated production and/or action of inflammatory cytokines can be detrimental and pathogenic. For example, it is well established that TNF plays a key role in RA pathogenesis and is a validated drug target of RA. Interestingly, in RA synovial fibroblasts, TNF induces expression of Notch1, Notch4, and Jagged2 as well as NICD nuclear translocation, a hallmark of Notch pathway activation (Ando et al., 2003). Moreover, in osteoclast precursors, Notch-RBP-J signaling is activated by TNF and in turn inhibits osteoclastogenesis and attenuates TNF-mediated inflammatory bone resorption in a feedback manner (Zhao et al., 2012). Another example of TNF-induced Notch activation is observed in a mouse pancreatic cancer model where TNF promotes expression of Notch target genes Hes1 and Hey1 (Maniati et al., 2011). Thus, TNF appears to function as an activator of Notch signaling in several cell types. IL-1β is another important pro-inflammatory cytokine. It is reported that IL-1β induces Notch target gene Hes1 expression in chondrocytes via Notch1 activation, suggesting that similar to TNF, IL-1β also has the potential to serve as Notch activator (Ottaviani et al., 2010). In addition to the prototypical pro-inflammatory cytokines such as TNF and IL-1β, TGFβ has also been shown to directly induce Hes1 expression in several cell types (Ostroukhova et al., 2006), expanding the panel of Notch-activating cytokines to include the anti-inflammatory/pleiotropic family. While many cytokines positively regulate Notch signaling and its target gene expression, interferon-γ (IFNγ) functions as a negative regulator of Notch pathway activation. In human primary macrophage, IFNγ drastically suppresses induction of canonical Notch target genes by TLR ligands and by Notch ligands (Hu et al., 2008). The precise mechanisms by which IFNγ antagonizes Notch signaling remain poorly defined and represents an interesting topic for future investigation.

### Molecular mechanisms of Notch activation by inflammatory stimuli

Notch target gene expression can be activated in myeloid cells by a wide array of inflammatory stimuli including TLR ligands and cytokines. However, the molecular mechanisms of such activation are ill defined. One attractive candidate pathway that could potentially mediate activation of Notch target genes in myeloid cells is NF-κB signaling that is activated by both TLR ligands and inflammatory cytokines and has been shown to interact with the Notch pathway in many systems such as cancer (Espinosa et al., 2010). Indeed, TLR and TNF-induced Notch target gene expression is often dependent on inhibitor of NF-κB kinases (IKKs) (Hu et al., 2008; Maniati et al., 2011), kinases required for NF-κB activation by inflammatory stimuli. Another group of signaling molecules implicated in mediating Notch pathway activation are mitogen-activated protein kinases (MAPKs) (Hu et al., 2008; Zeng et al., 2005), a family of serine/threonine protein kinases many of which are key regulators of inflammation. At least three distinct yet complementary mechanisms have been described to explain NF-κB-mediated activation of canonical Notch target genes: (1) Transcription factor cooperation. NICD has been shown to directly interact with NF-κB subunits and promotes transcription (Osipo et al., 2008). (2) Release of inhibitory molecules. For example, in resting cells inhibitor of NF-κB (IκB), which typically sequesters NF-κB in cytoplasm, was found to be present at the promoter regions of Hes1. Interestingly, TNF-induced Hes1 expression was thought to be associated with dismissal of IκBα from the Hes1 promoter (Aguilera et al., 2004). (3) Chromatin modification. TNF and TLR ligand-induced Hes1 gene transcription has been associated with upregulation of positive histone marks such as serine 10 phosphorylation and K14 acetylation of histone H3 at the Hes1 promoter (Hu et al., 2008; Maniati et al., 2011; Aguilera et al., 2004). Both IKKs and MAPKs have been implicated in mediating inflammatory signaling-induced chromatin modifications at the Notch target gene loci (Hu et al., 2008; Zeng et al., 2005; Aguilera et al., 2004). Taken together, NF-κB and MAPK signaling appears to play a critical role in mediating Notch target gene activation by inflammatory stimuli.

## REGULATION OF MYELOID DIFFERENTIATION BY NOTCH SIGNALING

**Figure 2:**
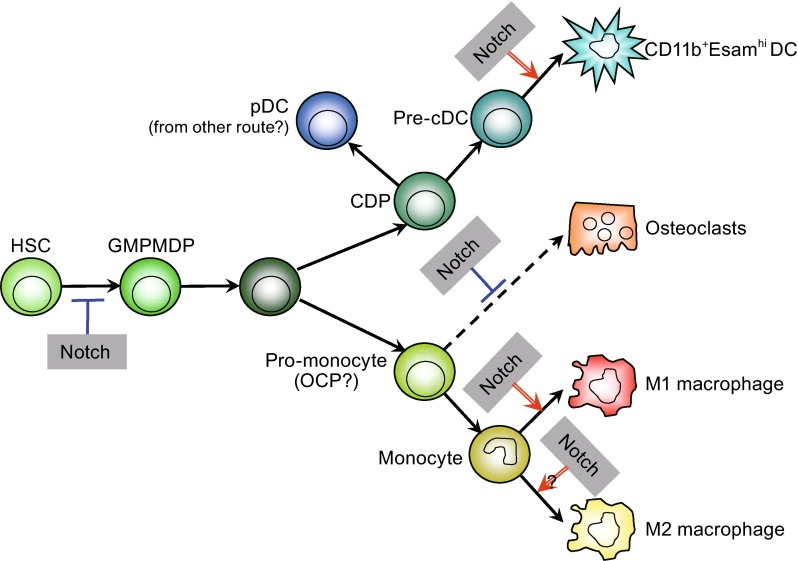
Regulation of myeloid cell development and differentiation by Notch signaling. Notch signaling critically controls multiple steps of myeloid cell development and differentiation program including early myelopoiesis, development of certain DC population, osteoclast differentiation, and inflammatory macrophage polarization. Abbreviations: HSC, hematopoietic stem cell; GMP, granulocyte-macrophage progenitor; MDP, macrophage-dendritic cell progenitor; CDP, common dendritic cell precursor; OCP, osteoclast precursor; pDC, plasmacytoid dendritic cells

The notion that inflammatory signals regulate Notch signaling and activate Notch target gene expression in myeloid cells has been increasingly appreciated as discussed above (Fig. 2). One obvious question is what are the roles of the Notch pathways in myeloid cell differentiation and function. In the following two sections, we will first discuss regulation of myeloid differentiation by Notch signaling under homeostatic conditions, and then the role of the Notch pathway in myeloid activation under immune and inflammatory conditions.

### Notch signaling and hematopoietic stem cells

Hematopoiesis is the developmental process, whereby pluripotent hematopoietic stem cells (HSCs) give rise to committed progeny that undergo proliferation and differentiation in response to both positive and negative soluble and cell-bound factors and cytokines, resulting in the continuous production of mature blood cells of various lineages. In the developing immune system, the Notch signaling pathway regulates interactions between HSCs, which express all four Notch receptors, and bone marrow stromal cells, which express various Notch ligands (Bigas et al., 2010). In this section, we will discuss recent progress in understanding the role of the Notch pathway in development and differentiation of myeloid cells. Although Notch signaling is thought to play a key role in myeloid cell differentiation from HSCs, there are discrepancies as to the mechanisms involved. One body of evidence demonstrates a role for Notch in the maintenance of progenitor cells and block of terminal differentiation of myeloid cells. In support of this hypothesis, retroviral transduction of the activated intracellular domain of Notch1 (NICD1) in 32D myeloid progenitor cells inhibited differentiation of mature granulocytes in response to granulocyte colony-stimulating factor (G-CSF), but not granulocyte macrophage colony stimulating factor (GM-CSF), without affecting proliferation of undifferentiated cells (Milner et al., 1996; Bigas et al., 1998). NICD2 inhibited differentiation of 32D cells in response to GM-CSF but not G-CSF (Bigas et al., 1998). These findings suggested that although both Notch1 and Notch2 inhibited myeloid differentiation, they may have distinct functions in HSCs depending on the specific differentiation signal involved. The Notch RAM domain, which contains the RBP-J binding region, was subsequently shown to be required for these Notch-mediated functions (Tan-Pertel et al., 2000), implying that Notch signals through the canonical RBP-J-dependent pathway to inhibit terminal differentiation and enhance survival of 32D myeloblast cells. Over-expression of the downstream RBP-J target Hes1 resulted in a similar phenotype (Tan-Pertel et al., 2000; Kumano et al., 2001). In addition, NICD1 or Hes1 expression blocked erythroid differentiation (Kumano et al., 2001; Lam et al., 2000) and Notch1 inhibition using a loss-of function approach resulted in spontaneous erythroid maturation (Lam et al., 2000). Promotion of HSC self-renewal and differentiation inhibition was also observed in response to various Notch ligands (Varnum-Finney et al., 1998; Carlesso et al., 1999; Varnum-Finney et al., 2003; Han et al., 2000).

On the other hand, Notch has been shown to be required for differentiation of mature myeloid cells. For example, Schroeder et al. (2000) demonstrated that conditional expression of NICD1 in 32D cells enhanced granulocyte differentiation and decreased self-renewal. Experiments using NICD1 deletion mutants indicated a role for the RAM domain in this process, and either Jagged1 stimulation or expression of a transcriptionally active form of RBP-J in 32D cells also promoted myeloid differentiation (Schroeder and Just, 2000).

Recently Klinakis et al. (2011) outlined a role for Notch signaling during early HSC differentiation *in vivo*. Inactivation of Notch signaling by targeting the γ-secretase complex member Nicastrin in mouse HSCs resulted in an aberrant accumulation of granulocyte/monocyte progenitors in peripheral blood, spleen and liver, diagnostic of the induction of chronic myelomonocytic leukemia (CMML)-like disease. Gene expression analysis revealed that Notch signaling regulates a myelomonocytic-specific gene signature through the suppression of gene transcription by Hes1. Further, somatic mutations were identified in multiple Notch pathway genes, including those that encode Nicastrin, MAML1 and Notch2, in samples from CMML patients, demonstrating a tumor-suppressive role for Notch signaling in addition to involvement in early HSC differentiation (Klinakis et al., 2011).

### Notch signaling and dendritic cell differentiation

In recent years, there has been much interest in the role of Notch signaling during the development of DCs (Cheng and Gabrilovich, 2008), which represent the key professional antigen presenting cells involved in the immune response to pathogens, tumor cells, and self antigens. DCs sample the environment in tissues and lymphoid organs and recognize pathogen-associated molecular patterns (PAMPs) by means of PRRs. Pathogen recognition and capture triggers a cascade of signaling events that leads to DC maturation, which involves outgrowth of dendrites, increased expression of MHC class II and co-stimulatory molecules, secretion of cytokines such as IL-12 and migration into T cell areas of lymphoid organs to present peptide antigen to naïve T cells. Thus, DCs are intimately involved in linking the innate and adaptive arms of the immune system.

There are two major subclasses of DCs, including conventional DCs (cDCs), which differentiate from myeloid progenitors in the bone marrow and plasmacytoid DCs (pDCs), which can arise from cells of both myeloid and lymphoid origin and specialize in virus recognition and secretion of type I interferon (IFN). cDCs are further classified according to their surface expression of specific myeloid markers. The more abundant CD8^−^CD11b^+^ DCs reside in the marginal zone of the splenic lymphoid follicles, a structure that filters incoming blood, and preferentially present exogenous antigens on MHC class II protein to CD4 helper T cells. CD8^+^CD11b^−^ DCs, on the other hand, are thought to mainly reside in T cell zones of the spleen, and present antigen via MHC class I to cytotoxic T cells (Sathe and Wu, 2011).

A potential role for Notch in the development of cDCs has been suggested by various *in vitro* studies (Ohishi et al., 2001; Weijzen et al., 2002; Cheng et al., 2003; Cheng et al., 2007; Zhou et al., 2009; Sekine et al., 2009; Cheng et al., 2001; Mizutani et al., 2000). Differentiation of mature DCs expressing MHC class II molecules in response to GM-CSF and IL-4 stimulation was decreased in HPCs from anti-sense Notch1 transgenic mice compared to cells from control mice (Cheng et al., 2001). Transduction of the anti-sense Notch1 HPCs with a constitutively active Notch1 almost completely restored the differentiation ability (Cheng et al., 2001). Studies from the same laboratory later strengthened these observations by demonstrating that DC differentiation was inhibited in embryonic stem cells and HSCs from Notch1-deficient mice (Cheng et al., 2003). Additionally, stimulation of primary murine peripheral blood monocytes with immobilized DLL1 inhibited macrophage development but permitted differentiation into DCs (Ohishi et al., 2001) and DLL1 may exert its effect on DC differentiation via activation of the Wnt signaling pathway (Zhou et al., 2009). In addition to the role of DLL1 in the development of differentiated DC populations, other Notch ligands participate (Sekine et al., 2009) and it has been demonstrated that Jagged1 induced DC differentiation in human monocytes (Weijzen et al., 2002). Further studies demonstrated that individual Notch ligands can differentially regulate DC differentiation. DLL1-expressing fibroblasts co-cultured with HPCs induced DC differentiation, whereas Jagged-1 expressing fibroblasts inhibited DC differentiation and promoted accumulation of immature myeloid cells (Cheng et al., 2007). The distinct effects of the various Notch ligands may reflect their physiological functions in the body as there is differential expression of Notch ligands in bone marrow and splenic stroma (Cheng and Gabrilovich, 2008).

In contrast to these findings, initial *in vivo* experiments demonstrated that although conditional knockout of Notch1 blocked T cell development in mice, myeloid development was not affected (Radtke et al., 1999; Radtke et al., 2000). However, it is possible that normal DC development in the absence of Notch1 is due to the potential redundancy of individual Notch receptors. As deletion of RBP-J is thought to recapitulate the phenotypes of individual receptor knockouts and of dominant-negative inhibition of Notch signaling, Caton et al. (2007) investigated the function of canonical Notch-RBP-J signaling using mice with a specific deletion of RBP-J in the DC compartment. They demonstrated that Notch signaling was essential for DC homeostasis in the spleen, in particular for survival and persistence of splenic CD8^−^CD11b^+^ DCs in the marginal zone, as evidenced by reduced cell survival and increased turnover in RBP-J-deficient mice. Other subsets of splenic DCs and DCs in the lymph nodes and tissues were not affected by RBP-J deletion and the selective requirement for Notch signaling in the CD8^−^CD11b^+^ DCs correlated with the specific and RBP-J-dependent expression of the Notch target gene Dtx1 (Caton et al., 2007). This group subsequently identified the Notch receptor involved in this phenotype and has demonstrated a key role for Notch2 (Lewis et al., 2011). Specific deletion of Notch2 in the DC compartment resulted in decreased numbers of CD11b^+^ DCs. Within this cell subset, blockade of Notch signaling ablated a distinct population characterized by high expression of the adhesion molecule ESAM, which is expressed on endothelium and regulates neutrophil extravasation. In support of these findings, NICD over-expression increased ESAM expression on CD11b^+^ DCs. Additionally Notch2 deletion led to the loss of CD11b^+^CD103 DCs in the intestinal lamina propria (Lewis et al., 2011). Consistent with the earlier findings using global Notch1 deletion (Radtke et al., 2000), specific deletion of Notch1 in DC populations was found to be dispensable for splenic DC development. Taken together, these findings imply a role for canonical Notch-RBP-J signaling in the development of tissue-specific DCs in the spleen and intestine and indicate that Notch2 is the specific receptor involved in these processes.

pDCs are phenotypically and functionally different from cDCs. Both positive and negative effects of Notch signaling on pDC development have been reported. In one study, Notch signaling via DLL1 was shown to support pDC formation from human HSCs (Olivier et al., 2006). A γ-secretase inhibitor (GSI) blocked this effect (Olivier et al., 2006). However, DLL1 blocked pDC development from early thymic precursors (Dontje et al., 2006). Inactivation of Notch1 did not affect the development of pDCs, suggesting that this receptor is not essential for differentiation of this cell subtype (Radtke et al., 2000; Lewis et al., 2011; Ferrero et al., 2002).

The reasons for the differences in the various reports outlined above are unclear. Experimentally, different constructs and protein expression systems, mouse models and Notch ligands (soluble or immobilized) were used. Moreover, it is widely accepted that Notch signaling is highly cell context specific and in the hematopoietic microenvironment, the effects of Notch activation on HSCs are likely to be influenced by growth factors, cytokines and cross-talk with other signaling pathways that tailor the developmental cell fate. Physiological stimulation of Notch activity *in vivo* is likely to be transient in nature. Additionally, although RBP-J plays a key role in canonical Notch signaling, Notch can signal independently of RBP-J and RBP-J can be activated by alternative signaling pathways (Martinez Arias et al., 2002). While the precise molecular mechanisms of Notch-mediated regulation are far from fully understood, it is clear that Notch signaling influences differentiation of specific myeloid subsets in a cell- and ligand-specific context.

### Notch signaling and osteoclastogenesis

Physiological bone development and remodeling represents a balance between bone formation by osteoblasts and bone resorption mediated by osteoclasts, which are multinucleated cells derived from the monocyte-macrophage lineage. Osteoclast differentiation is a multi-step process that culminates in expression of the osteoclast marker TRAP (tartrate-resistant acid phosphatase), multinucleation and bone-resorping activity. Osteoclastogenesis depends on differentiation signals from stromal cells and synovial fibroblasts, and is physiologically triggered by RANKL (receptor activator of NF-κB ligand) in the presence of M-CSF and other co-stimulatory factors. Recruitment of these resorptive cytokines can be physiologically restricted by osteoprotegerin (OPG, also known as osteoclastogenesis inhibitory factor) (Zhao and Ivashkiv, 2011). RANKL stimulation of osteoclast precursors leads to the induction of cell signaling cascades resulting in activation of the master transcriptional regulator of osteoclastogenesis, NFATc1 (nuclear factor of activated T cells, cytoplasmic 1). Numerous inflammatory molecules, such as TNFα, IL-1β, IL-17, and TLR ligands, promote osteoclastogenesis in synergy with RANKL to induce pathological bone resorption in inflammatory settings. As such, osteoclasts have been implicated in musculoskeletal tissue damage and the pathogenesis of diseases characterized by inflammatory osteolyis, including RA, psoriatic arthritis, and peridontitis. In these disease settings, abnormally enhanced osteoclast formation and activity causes bone loss that results in pain, deformity, osteopenia, osteoporosis and even fracture. The extent of bone destruction in inflammatory disease is determined by the balance between positive and negative regulators of osteoclastogenic factors (Zhao and Ivashkiv, 2011).

Notch signaling has been implicated in osteclastogenesis during normal bone homeostasis and inflammation. Notch receptors, ligands and target genes have been detected in osteoclast precursors and differentiated osteoclasts (Yamada et al., 2003; Bai et al., 2008; Fukushima et al., 2008). A role for Notch in promoting osteoclast differentiation has been described. Suppression of Notch signaling by GSI treatment or shRNA for Notch2 inhibited RANKL-induced osteoclast differentiation (Fukushima et al., 2008), whereas activation of Notch signaling by stimulation with Jagged1 or NICD2 over-expression increased NFATc1 promoter activity and promoted osteoclastogenesis (Fukushima et al., 2008). However, the remaining body of evidence investigating Notch signaling during osteoclast development has indicated a suppressive role for Notch. Firstly, Yamada et al., demonstrated that immobilized DLL1 inhibited osteoclast differentiation from mouse bone marrow cells in response to RANKL and M-CSF (Yamada et al., 2003). DLL1 also decreased surface expression of the M-CSF receptor c-Fms on the bone marrow cells. Stromal cells over-expressing NICD1 reduced M-CSF production and enhanced RANKL and OPG production, resulting in the decreased capability of these cells to support osteoclastogeneis (Yamada et al., 2003). Subsequently, genetic approaches indicated that deletion of Notch1 or combined Notch1–3 enhanced osteoclastogenesis in response to M-CSF or RANKL, resulting in increased resorptive activity (Bai et al., 2008). Osteoclast precursors with inactivated Notch1–3 exhibited increased expression of c-Fms. Overexpression of NICD1 or Jagged1 stimulation of wild type BMDMs blocked their differentiation into osteoclasts in response to M-CSF and RANKL (Bai et al., 2008).

Further studies have supported an inhibitory role for Notch in the context of TNFα-induced osteoclastogenesis in the inflammatory setting (Zhao et al., 2012). RBP-J was shown to strongly repress TNF-induced osteoclastogenesis, as myeloid specific deletion of RBP-J dramatically increased osteoclastogenesis and resulted in severe bone destruction in a TNF-induced inflammatory bone resorption model. Additionally, knockdown of RBP-J expression in human osteoclast precursors by RNAi enhanced TNF-induced osteoclast differentiation. By activating RBP-J using forced expression of NICD1 in myeloid osteoclast precursors, TNF-induced inflammatory bone resorption was dramatically decreased. RBP-J was demonstrated to suppress induction of NFATc1 by attenuating cFos activation and inhibiting induction of Blimp1, thereby preventing the downregulation of transcriptional repressors such as IRF8 that block osteoclast differentiation (Zhao et al., 2012). Such inhibitory effects are possibly attributed to Notch-mediated crosstalk with other pathways such as immunoreceptor tyrosine-based activation motif-containing (ITAM-containing) receptors and adaptors (Li et al., 2014) as well as TAK1 signaling (Swarnkar et al., 2015). Thus, the majority of studies have delineated a direct inhibitory role for Notch signaling in the physiological context of osteoclastogenesis and inflammatory bone resporption. In addition, Notch signaling may indirectly regulate osteoclast differentiation *in vivo* by regulating the differential expression of RANKL and OPG on osteoblast lineage cells (Hilton et al., 2008; Engin et al., 2008; Zanotti and Canalis, 2010).

## REGULATION OF MYELOID ACTIVATION AND FUNCTION BY NOTCH SIGNALING

Besides its role in myeloid cell differentiation, recently there is increasing evidence supporting a role for Notch signaling in regulating activation and function of terminally differentiated myeloid cells. As regulation of acute activation by the Notch pathway is still an emerging concept in the field, there are controversies regarding the exact roles and mechanisms of action of this pathway. We will summarize the current knowledge regarding regulation of activation and function of myeloid cells by Notch signaling below.

### Notch signaling in DC function

Accumulating evidence has indicated that Notch signaling influences both functional DC maturation and DC-mediated T cells responses. Notch receptors are expressed on T cells and Notch ligands are expressed on DCs (Cheng and Gabrilovich, 2008). As mentioned above, pathogen recognition triggers DC activation and presentation of peptide antigen to naïve T cells, thus expression of surface molecules on DCs specifies the T cell response. Th1 cells are characterized by production of IFNγ and are mainly involved in cellular immunity against intracellular pathogens. On the other hand, Th2 cells play a role in immunity against extracellular pathogens. Indeed, certain findings support a ligand-specific role for DC-expressed Notch ligands in specifying various T cell lineages (Amsen et al., 2004; Skokos and Nussenzweig, 2007; Ito et al., 2009; Maekawa et al., 2003; Hoyne et al., 2000; Schaller et al., 2007). Further, the ESAM high DC population identified by Lewis et al., whose development depended on canonical Notch2-RBP-J signaling, was required for optimal T cell priming (Lewis et al., 2011), as evidence by decreased T cell proliferation in the spleens of RBP-J-deficient mice that specifically lacked the ESAM high subset. Additionally, Notch2 provided a tissue-specific developmental signal for the CD11b CD103 DC population in the small intestine and colon, which supported optimal differentiation of Th17 cells, a major effector CD4 population in this tissue type (Lewis et al., 2011). Notch2 induction in IL-19-mediated regulation of lung DC maturation has been demonstrated and this could have potential implications for antigen presenting cells involved in autoimmune disease, as IL-19 has been reported to enhance chronic inflammation associated with asthma, psoriasis and rheumatoid arthritis (Hoffman et al., 2011). However, there are also reports suggesting lack of a role of Notch signaling in specifying Th cell fate especially Th1 versus Th2 differentiation (Ong et al., 2008). The precise involvement of Notch in Th differentiation remains to be sorted out possibly awaiting experiments utilizing genetic fate mapping tools.

RBP-J has been shown to play a critical role in the maturation of peptide or LPS-induced DCs (Weijzen et al., 2002; Wang et al., 2009). Two hallmarks of DC maturation, namely dendrite outgrowth and MHC class II expression, were significantly reduced in RBP-J-deficient DCs during LPS-mediated maturation (Wang et al., 2009). Additionally, RBP-J-deficient DCs stimulated significantly weaker T cell proliferation than control cells. Pathogen-mediated DC maturation is associated with chemokine receptor expression and LPS-mediated CXCR4 expression was decreased in RBP-J-deficient DCs. Over-expression of CXCR4 rescued the maturation defects of RBP-J-deficient DCs by restoring dendrite outgrowth and MHC II expression. Activation of Notch signaling using DLL1 upregulated surface expression of CXCR4 and promoted DC maturation, and these findings were reversed using a GSI (Wang et al., 2009).

Further studies from the Han laboratory outlined a role for Notch signaling in the anti-tumor function of DCs. Loss of RBP-J in DCs impaired DC-dependent anti-tumor responses (Feng et al., 2010). RBP-J-deficient DCs were unable to repress tumor growth when co-injected with tumor cells in mice and their capacity to recruit T cells to solid tumors and draining lymph nodes was compromised. In addition, RBP-J-deficient DCs exhibited attenuated expression of the antigen presenting molecules MHC II, co-stimulatory molecules CD80 and CD86 in response to tumor antigens and displayed a reduced capacity to activate T cells in relation to T cell proliferation, T cell cytokine production (IFNγ and IL-4) and cytotoxicity (Feng et al., 2010). Taken together, these results demonstrate clear involvement of Notch-RBP-J signaling in DC maturation and in the execution of DC-mediated T cell activation in the setting of both infection and tumor immunity.

### Notch signaling in macrophage activation

Macrophages are versatile cells with diverse functions in inflammation, tissue remodeling, angiogenesis and tumor immunity. They respond to a wide variety of environmental cues to regulate immunity and inflammation by sensing microbial pathogens, secreting cytokines and inflammatory mediators and presenting antigen to T cells. Similar to DCs, macrophages distinguish pathogens and self antigens through PRRs of the TLR, NLR, and RLR families. Upon PAMP recognition, TLRs engage TIR-containing adaptor molecules and kinases that trigger signaling pathways resulting in the activation of transcription factors, including NF-κB, IFN-regulatory factors (IRFs) and AP-1. This leads to the induction of proinflammatory cytokines such as IL-1, TNFα, IL-6, type I (α and β) IFNs, and immunoregulatory cytokines including IL-12 and IL-10. Unrestrained activation of TLR signaling can lead to excessive inflammation and tissue damage and contribute to sepsis, chronic inflammation, autoimmune disease and cancer. In addition to TLR-mediated PAMP recognition, macrophages can also be activated by IFNγ stimulation. IFNγ synergizes with TLRs to induce augmented production of inflammatory cytokines (Hu and Ivashkiv, 2009).

**Figure 3:**
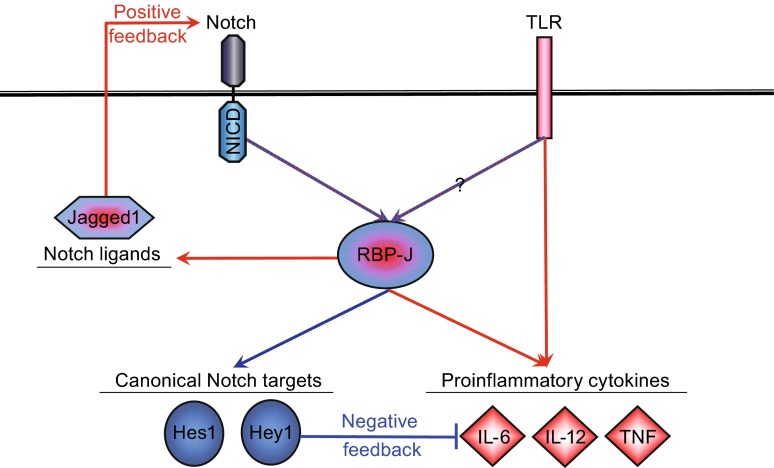
Crosstalk between the TLR signaling pathway and the Notch pathway. Expression and/or function of various components of the Notch pathways could be regulated by TLR signaling. Conversely, Notch pathway components positively or negatively modulate TLR-activated transcriptional, translational, and metabolic programs to finetune outcomes of immune responses

Recent studies have delineated a role for canonical Notch signaling during macrophage activation (Fig. 3). Indeed, constitutive expression of Notch pathway components has been detected on primary macrophages and established macrophage cell lines of both human and mouse origin (Zhang et al., 2010; Fung et al., 2007; Foldi et al., 2010; Monsalve et al., 2006; Hu et al., 2008). Notch receptor, ligand and target gene expression can be further enhanced in macrophages in response to proinflammatory stimuli, including various TLR ligands (Fung et al., 2007; Ito et al., 2009; Foldi et al., 2010; Monsalve et al., 2006; Monsalve et al., 2009; Palaga et al., 2008; Hu et al., 2008; Tsao et al., 2011; Outtz et al., 2010; Goh et al., 2009), activated lymphocyte-derived DNA (ALD-DNA) (Zhang et al., 2010), influenza infection (Ito et al., 2011), *Mycobacterium bovis* Bacille Calmette-Guérin (*M. bovis* BCG) infection (Narayana and Balaji 2008; Bansal et al., 2009; Kapoor et al., 2010) or stimulation with helminth antigens (Goh et al., 2009).

Numerous lines of evidence from studies employing genetic or RNAi-mediated disruption of Notch pathway components, inhibition of Notch receptor cleavage using GSIs or gain-of-function studies expressing constitutively active NICD, support a positive role for canonical Notch signaling during the inflammatory response in activated macrophages. Firstly, over-expression of NICD1 in Raw264.7 cells increased STAT1 activation and STAT1-dependent transcription in response to LPS and IFNγ, leading to higher expression of molecules characteristic of mature activated macrophages (Monsalve et al., 2006; Monsalve et al., 2009). Secondly using a similar gain-of-function approach, Raw264.7 cells over-expressing NICD1 exhibited increased expression of the cytokines TNFα and IL-6 and the enzyme iNOS (Monsalve et al., 2009). NICD1 expression also upregulated both basal and LPS-induced NF-κB activation, as demonstrated by increased phosphorylation and degradation of IκBα, increased nuclear translocation of NF-κB and enhanced binding of NF-κB subunits to the TNFα and iNOS promoters. Inhibiting Notch signaling with the GSI DAPT or shRNA for Notch1 abrogated NF-κB activity following LPS stimulation (Monsalve et al., 2009). Thus, one potential mechanism whereby Notch signaling contributes to the inflammatory response is by enhancing NF-κB signaling.

In support of these findings, Notch signaling inhibition in primary BMDMs using another GSI, IL-CHO, decreased LPS plus IFNγ-mediated induction of IL-6, iNOS, and TNFα expression (Palaga et al., 2008). In addition, both IL-CHO or siRNA for Notch1 decreased translocation of NF-κB into the nucleus upon stimulation with LPS/IFNγ in Raw264.7 cells (Palaga et al., 2008). Treatment with the GSI DAPT was shown to attenuate LPS-mediated IL-1β and IL-6 in Raw264.7 cells and decrease the levels of these cytokines in an *in vivo* sepsis model (Tsao et al., 2011). These results were confirmed and extended by our laboratory using a rigorous genetic approach demonstrating that deletion of RBP-J in the myeloid compartment attenuated TLR-induced expression of key inflammatory mediators including TNFα, IL-6, IL-12, and iNOS (Hu et al., 2008; Xu et al., 2012). Mechanisms of Notch-regulated macrophage gene expression may involve: 1) Direct binding of RBP-J to gene promoters supported by observations that a mutation in the putative RBP-J element in the IL-6 promoter diminished LPS-driven IL-6 reporter gene activity (Hu et al., 2008). 2) Indirect regulation via promoting translation of transcription factors required for gene activation such as IRF8 (Xu et al., 2012). 3) Reprogramming mitochondria metabolic status to link Notch signaling with metabolic pathways (Xu et al., 2015). Functionally, RBP-J deficiency protected mice from lethality following endotoxin challenge (Hu et al., 2008) and comprised host defense to bacterial pathogens *in vivo* (Xu et al., 2012). Furthermore, RBP-J-deficient macrophages displayed attenuated capacity to activate T cells (Wang et al., 2010). Thus, Notch signaling via the canonical Notch-RBP-J pathway contributes to TLR-induced cytokine gene expression during inflammatory macrophage activation.

Consistent with this hypothesis, involvement of Notch signaling in the inflammatory response in the context of wound healing has been described by Outtz et al. (2010). Decreased macrophage recruitment and TNFα expression was observed in wounds of mice with myeloid-specific inhibition of Notch1 compared to wild type controls. Experiments incorporating GSIs or NICD1 over-expression demonstrated that canonical Notch signaling mediates LPS/IFNγ-induced VEGFR-1 expression, which is important for the recruitment and function of macrophages during angiogenesis and inflammation. Similar to the studies outlined above, LPS/IFNγ-mediated cytokine induction was attenuated in Notch1-deficient macrophages (Outtz et al., 2010). In an experimental model for systemic lupus erythematosus (SLE), blocking Notch signaling using the GSI DAPT attenuated induction of inflammatory cytokines and cell surface markers in Raw264.7 cells treated with ALD-DNA (Zhang et al., 2010). GSI treatment also inhibited the antigen presenting capabilities of ALD-DNA-stimulated BMDMs (Zhang et al., 2010), strengthening the role for the Notch-RBP-J pathway during the inflammatory response.

Notch1 has been implicated in *M. bovis* BCG infection-mediated induction of Suppressor of cytokine signaling (SOCS) 3, which is a critical negative regulator of cytokine signaling (Narayana and Balaji, 2008). GSI-I treatment inhibited SOCS3 induction by *M. bovis* BCG in mouse peritoneal macrophages. Similar results were obtained using siNotch1 in Raw264.7 cells. NICD1 over-expression potentiated SOCS3 induction in response to *M. bovis* BCG. This group subsequently outlined a role for Notch signaling in *M. bovis* BCG-mediated up-regulation of cyclooxygenase-2 (COX2) (Bansal et al., 2009) and matrix metalloproteinase-9 (MMP9) (Kapoor et al., 2010), an effector molecule that participates in cell motility during inflammatory responses. Raw264.7 cells over-expressing NICD1 exhibited increased COX2 and MMP9 induction in response to *M. bovis* BCG infection, while siNotch1 or GSI-I treatment blocked this effect. Increased expression of NICD1, Hes1 and MMP9 was detected in brain tissue samples from patients with tuberculosis meningitis (Kapoor et al., 2010). These studies provide insight into the role of Notch signaling in host-mycobacteria interactions.

In contrast with the findings described above, Zhang et al., have described the inhibitory role for Notch during TLR-mediated inflammatory responses in macrophages (Zhang et al., 2012). TLR-mediated induction of IL-6 and TNFα was reduced upon over-expression of NICD1 or NICD2 in mouse peritoneal macrophages (Zhang et al., 2012). Induction of the anti-inflammatory cytokine IL-10 was enhanced in this experimental model. Loss-of-function experiments where Notch1 expression was inhibited using siRNA or GSI X treatment, led to increased TNFα and IL-6 production and decreased IL-10. Notch signaling was suggested to exert the observed inhibitory effects by attenuating TLR-mediated ERK phosphorylation and NF-κB transcriptional activity (Zhang et al., 2012). Importantly, in the context of tumor immunity, depletion of RBP-J in tumor associated macrophages (TAMs) compromises TAM differentiation and function and restores tumor-infiltrating cytotoxic T cell responses (Franklin et al., 2014), suggesting a suppressive role of Notch signaling in anti-tumor immune effector functions.

Several possible reasons may explain the discrepancies between these reports and the previous work outlining a positive role for Notch signaling during TLR-mediated macrophage activation. Different GSIs were used in the various studies including DAPT (Monsalve et al., 2009; Tsao et al., 2011), IL-CHO (Palaga et al., 2008), GSI-I (Narayana and Balaji 2008; Bansal et al., 2009) GSI IX (Wang et al., 2010) or GSI X (Tsao et al., 2011) and GSIs have been shown to affect signaling pathways in addition to Notch (Hass et al., 2009). Additionally, different cell models from different species were used in the various studies, namely mouse Raw264.7 cells, mouse BMDMs and peritoneal macrophages, rat primary alveolar macrophages or human PBMC-derived macrophages. The specific outcome of simultaneous Notch and TLR stimulation in macrophages is likely to involve a tightly regulated balance between positive and negative regulatory signals in a cell context and Notch ligand-specific fashion. Further studies into global Notch-RBP-J target identification using these approaches in innate immune cells will provide valuable insights into the functional role of Notch signaling in the inflammatory setting.

### Notch signaling in other cell types

The skin epidermis represents a physical barrier that protects against infectious agents and mechanical injury. Keratinocytes are the main epithelial cell type that generates the epidermal layer of the skin. Epidermal homeostasis and barrier integrity is maintained through the coordinated regulation of proliferation, migration and cell death. During epidermal inflammation, homeostasis collapses resulting in the production of alarmins by keratinocytes and the recruitment of immune cells to the site of tissue damage. One such alarm signal is thymic stromal lymphoprotein (TSLP), which is an IL-7-like cytokine produced by epithelial cells and is considered a general biomarker for skin barrier defects. TSLP expression is sustained as long as barrier defect persists, thus TSLP-mediated activation of dermal innate immune cells and the subsequent local cellular immune response is thought to contribute to the pathogenesis of eczema/atopic dermatitis (AD) (Blanpain et al., 2006).

Notch signaling has been demonstrated to sustain the epidermal barrier by supporting keratinocyte terminal differentiation (Blanpain et al., 2006; Rangarajan et al., 2001). Furthermore, emerging evidence suggests an important role for Notch in preventing inflammatory skin disease and maintaining epidermal homeostasis. The ablation of canonical Notch signaling in keratinocytes by simultaneous deletion of both Notch1 and Notch2 or RBP-J resulted in defective skin barrier function and increased expression of TSLP, leading to the development of a severe AD-like skin phenotype in mice (Demehri et al., 2008; Dumortier et al., 2010). Interestingly, the AD-like inflammation associated with Notch deficiency was accompanied by G-CSF-induced myeloproliferative disorder (MPD) characterized by an increase in immature myeloid populations in the bone marrow and spleen (Dumortier et al., 2010), suggesting a protective role for Notch against myeloproliferation similar to that observed by Klinakis et al. (2011).

Individuals with severe AD are at increased risk of developing the chronic lung disease allergic asthma, a progression termed atopic march (Demehri et al., 2008). Demehri et al., demonstrated that AD-like disease resulting from deletion of RBP-J in the skin predisposed mice to allergic asthma in an ovalbumin model of allergic inflammation. As RBP-J was not deleted in the lung, these data show that the skin barrier defect served as a primary risk factor for development of asthma in the normal lung (Demehri et al., 2008). TSLP was required for asthma susceptibility in animals with the AD-like pathology, as deletion of the TLSP receptor in RBP-J-deficient mice blocked the progression from allergic skin inflammation to asthma. Using a gain-of-function approach, TSLP overexpression in epidermal keratinocytes conferred an asthmatic phenotype (Demehri et al., 2008). These findings indicate that Notch-RBP-J-mediated suppression of TSLP prevents inflammatory skin disease and the associated risk of progression to allergic asthma.

Murthy et al., have provided additional evidence for Notch-mediated regulation of skin barrier immunity by investigating the role of the protease ADAM17 (also called TACE) (Murthy et al., 2012). As mentioned earlier, ADAM-mediated cleavage of Notch receptors is a one of the key steps in the activation of Notch signaling (Kopan and Ilagan, 2009). Similar to simultaneous ablation of Notch1 and Notch2 or RBP-J (Dumortier et al., 2010), ADAM17 inactivation in the keratinocyte compartment resulted in increased epidermal TSLP expression and spontaneous onset of AD and MPD in mice (Murthy et al., 2012). Ectopic activation of Notch rescued local skin inflammation and MPD in ADAM17-deficient mice. Notch was shown to inhibit AP-1-mediated induction of TSLP by antagonizing recruitment of the AP-1 subunit c-Fos to the *TSLP* promoter (Murthy et al., 2012). This observation that Notch inhibits AP-1 activity is in keeping with studies by Monsalve et al., demonstrating decreased AP-1 driven transcriptional activity in response to proinflammatory stimuli in macrophages over-expressing NICD1 (Monsalve et al., 2006; Monsalve et al., 2009).

ADAM17 was found to play a role in basal Notch activation in the adult epidermis as decreased levels of NICD and Notch target genes (*Hes5*, *Hey1*, and *Hey2*) were detected in keratinocytes from the ADAM17-deficient mice (Murthy et al., 2012). Although both ADAM10 and ADAM17 have been reported to cleave Notch receptors and facilitate NICD release by γ-secretase, there is controversy over the specific ADAM involved. Bozkulak et al., demonstrated that although Notch1 was a substrate for both ADAM10 and ADAM17, the specific protease required for NICD activation was context dependent, with ADAM10 absolutely required for Notch1 signaling in response to Notch ligands, while ligand-independent activation was regulated by ADAM17 (Bozkulak and Weinmaster, 2009). *In vivo* studies have shown that ADAM10 is essential for Notch2 activation (Gibb et al., 2010). Murthy et al., demonstrated that the ADAM17-mediated Notch activation in keratinocytes occurred in a ligand-independent manner (Murthy et al., 2012). NICD generation and Notch target gene expression in response to EDTA or increased calcium exposure was blocked in ADAM17-deficient keratinocytes but was unaffected in cells stimulated with the Notch ligand DLL4 (Murthy et al., 2012). The developmental context of Notch activation is likely to be facilitated by ADAM10, as keratintocyte development was inhibited in mice lacking epidermal ADAM10 (Weber et al., 2010). ADAM17 deletion on the other hand did not affect keratinoctye differentiation in this study (Weber et al., 2010). Murthy et al., propose a ligand-independent Notch signaling model, whereby ADAM17 controls barrier homeostasis and immunity in their experimental system, but not keratinocyte development (Murthy et al., 2012).

Taken together, these reports indicated that blocking Notch signaling leads to increased TSLP induction and AD in mice. In support of this hypothesis, expression of Notch receptors was downregulated in clinical skin samples from AD patients (Dumortier et al., 2010) and gene expression analysis of publicly available microarray data revealed differential expression of Notch2, Notch3 and presenelin1 in patients with AD and psoriasis (Murthy et al., 2012). The AD-like phenotype observed upon inactivation of Notch pathway components in mice keratinocytes was associated with asthma progression and MPD. Thus, Notch signaling plays a key role in maintenance of healthy epithelial barrier integrity and may decrease the risk of AD-associated MPD or asthma development.

## CONCLUSIONS

Due to the key role of Notch in these fundamental cell processes, dysregulated Notch signaling is associated with a number of human disorders, including developmental syndromes and cancer. More recently, polymorphisms in genes associated with Notch signaling have been linked to rheumatoid arthritis (Stahl et al., 2010), suggesting a previously unappreciated connection between Notch and autoimmunity. Although much remains to be learned about the role of Notch in inflammatory conditions, the rapidly accumulating body of literature strongly favors the notion that the Notch pathway is a critical regulator of innate immunity and inflammation. There is an emerging pattern of reciprocal regulation between Notch signaling and inflammation in that inflammatory stimuli activate myeloid Notch signaling and Notch signaling in myeloid cells in turn modulates inflammatory responses. As the Notch pathway is easily amenable to pharmacological manipulations such as γ-secretase inhibitors and metalloprotease inhibitors, targeting Notch signaling may represent a new and promising approach to modulating inflammation in relevant disease states. Moreover, availability of reagents that can target specific Notch receptors makes it possible to further focus the specificity of treatment (Wu et al., 2010). Indeed, therapies targeting the Notch pathway have shown efficacy in animal models of SLE and inflammatory arthritis (Park et al., 2015; Zhang et al., 2010; Sekine et al., 2012). Apparently, there might be a long way to go before the field appreciates the Notch pathway as a key regulator of inflammation among other well-established players and fully embraces the idea of Notch signaling in acute responses besides its role in immune cell development.
